# *Capparis sepiaria’s* root bark aqueous lyophilisate shows antiamnesic properties on scopolamine induce cognitive impairment in mice

**DOI:** 10.1016/j.ibneur.2023.10.009

**Published:** 2023-11-02

**Authors:** Francis Bray Yassi, Gwladys Temkou Ngoupaye, Tatiana Diebo Kom, Gabriella Dongmo Tonleu, Maxwell Blesdel Adassi, Aurelien Fossueh Foutsop, Elisabeth Ngo Bum

**Affiliations:** a Department of Biological Sciences, Faculty of Science, University of Ngaoundere, P.O. Box 454, Ngaoundere, Cameroon; b Department of Animal Biology, Animal Physiology and Phytopharmacology Research Unit, University of Dschang, P.O. Box 67, Dschang, Cameroon; c Department of Biological Sciences, Faculty of Science, University of Maroua, P.O. Box 814, Maroua, Cameroon

**Keywords:** *C. sepiaria*, Amnesia, Acetylcholinesterase, Oxidative stress

## Abstract

*Capparis sepiaria* (Capparaceae) is a plant used in African traditional medicine to treat psychiatic disorders. The aim of this study was to assess the anti-amnesic effect of aqueous lyophilisate of the root bark of *Capparis sepiaria* (*C. sepiaria*) on scopolamine-induced animal model of memory impairment using Swiss albino adult mice of both sexes. Memory integrity was assessed by Morris water Maze test**,** Novel Object Recognition (NOR) and Object-location memory (OLT) tasks were used to assess behavioural components of memory processes and learning. Malondialdehyde (MDA), reduced glutathione (GSH), NO levels and catalase were used to assess oxidative stress while acethylcholinesterase activity was used to evaluate acetylcholine activity in the hippocampus tissues. The quantitative phytochemistry and acute toxicity of the roots of *C. sepiaria* were also evaluated. The aqueous lyophilisate of *C. sepiaria* at doses of 10 mg/kg and 40 mg/kg significantly increased the discrimination index in the Morris Water Maze and the objet location tasks. The aqueous lyophilisate of *C. sepiaria* significantly increased hippocampal GSH and catalase levels and decreased hippocampal MDA, NO levels and achetylcholinesterase (AChE) activities. The aqueous lyophilisate of *C. sepiaria* showed no acute toxicity with a LD_50_ > 5000 mg/kg, and revealed a content of flavonoids, tannins and phenols. These results suggest that *C. sepiaria* improve memory impairment induced by scopolamine and therefore possess antiamnesic properties. These properties would result from a modulation of cholinergic neurotransmission as well as an antioxidant activity of the plant.

## Highlights

• *Capparis sepiaria* exhibits anti-amnesic properties in a model of scopolamine-induced cognitive deficits.
• *Capparis sepiaria* interact specifically with hippocampal-related tasks such as spatial memory as evaluated by the MWM and the OLT.
• *Capparis sepiaria* inhibits AChE activity in the hippocampus and enhances anti-oxidant mechanisms.
• *Capparis sepiaria* is non-toxic and is tolerated following an acute toxicity study.

## 1 Background

Cognitive impairment is a condition which affects approximately 50 million of people worldwide, and is most often characterized by a progressive decline of cognitive functions such as memory and thinking, as well as behaviour. Amnesia is a cognitive deficit characterized by profound memory loss, which is a syndrome of Alzheimer's disease (AD) (Johnston and Narayanasamy, 2016). Oxidative stress has been shown to be one of the underlying mechanism at the origin of memory deficits and behavioural abnormalities (Liu et al., 2017). As such, during oxidative stress, overproduction of Reactive oxygen species (ROS) and reactive nitrogen species (RNS), such as nitric oxide (NO) leads to the oxidation of lipids, proteins, cell membranes, and DNA, which often results in cell aging and damaged neurons in the hippocampus, causing impaired cognitive functions and memory (Fischer and Maier, 2015). Literature substantiates the close link between memory deficit and distorted cholinergic system. Indeed, it has been shown that damage of the nicotinic acetylcholine (Ach) receptors in the brain increases acetylcholinesterase (AChE) activity. This increase in AChE leads to a decrease in ACh’s brain concentration, thus impairing cognitive function (Ngoupaye et al., 2020a). One of the best known therapeutic strategy for AD dementia is the inhibition of AChE (Nazir et al., 2020). Scopolamine is a cholinergic antagonist known to interfere with the transmission of acetylcholine in the central nervous system. As such, it has been used as a reference inducing agent for animal models of amnesia (Ngoupaye et al., 2017). Its administration induces oxidative stress as well as memory and cognitive deficits in mice and rats (Nazir et al., 2020). To alleviate amnesia, currently available treatments target cholinergic synapses to increase synaptic acetylcholine (ACh) levels and relieve memory deficits (Liu et al., 2017).

Many treatments used against memory declines such as tacrine, and memantine have been shown to have some side effects such as nausea, vomiting, diarrhea, weight loss, indigestion and muscle weakness, dizziness, headache and hepatotoxicity (Korabecny et al., 2012, Matsunaga et al., 2015). Therefore, effective alternative treatments showing little or no side effects are encouraged.

*Capparis sepiaria* (*Capparis corymbosa*), called Wild caper-bush in England, is widespread in tropical Asia, Australia, and tropical Africa, where it is extensively used in traditional medicine (Lemmens, 2013). Its interesting antibacterial, anti-inflammatory, analgesic activity have already been approved in India (Satyanarayana et al., 2010), which corroborates the utilization of the plant in traditional medicine (Ennaceur et al., 1997). In Cameroon, the bark of the roots of *C. sepiaria* is utilized in traditional medicine to treat problems related to cognitive and mental disorders. This study focuses on the anti-amnesic properties of *C. sepiaria*’s aqueous lyophilisate on scopolamine-inducing memory loss in mice.

## 2 Material ad methods

### 2.1 Animals

Adult Swiss albino mice of both sexes (2– 2.5 months old) (n = 35) weighing 19–25 g were used. Animals were grouped in subgroup of 3 females and 4 males for a total number of 7 per group test. To avoid animals mating in each group we kept them separated according to the sex during the waiting time and the test period. The distribution was done according to Miller et al., 2017, and our previous studies, where this gender distribution was not having an influence in the responses, and has limited sexual dimorphism overall group effect (Foutsop et al., 2023, Ngoupaye et al., 2018, Ngoupaye et al., 2020a). We also considered previous studies that have shown that female are more sensitive than male mice to scopolamine effect on water maze performance (Berger-Sweeney et al., 1995, Giacobini and Pepeu, 2018) and that Ach released in the hippocampus is similar between male and female in light period (period used during our experiments) (Mitsushima, 2011; Giacobini and Pepeu, 2018). Treatment of animals and experimental procedure were in accordance with the guidelines of the Cameroonian bioethics committee (reg N°. FWA-IRB00001954) and with the NIH- Care and Use of Laboratory Animals manual. Accordingly, efforts were made to minimize animals' suffering and to decrease the number of animals used for the experiment. However, all animals were allowed to drink water ad libitum.

### 2.2 Plant collection and extraction

The roots of *Capparis sepiaria* were harvested at Lara, Kaele, Cameroon and later identified at the national herbarium of Cameroon in Yaoundé in comparison with voucher number (reference number 14194/SRF/Cam). A mass of 200 g of the root bark powder was introduced into 2 L of distilled water, macerated for 5 h, filtered using Whatman filter paper Nº1, the filtrate was then frozen and lyophilized at 0 °C. At the end of the process, a mass of 49 g of dry extract was obtained. The extraction yield was 24.5%.

### 2.3 Treatments

To assess the anti-amnesic properties of *C. sepiaria*, animals were grouped as follow: Group I was treated with distilled water and served as vehicle control. Group II was equally treated with distilled water + scopolamine and served as negative control. Group III received tacrine an inhibitor of acetylcholinesterase (10 mg/kg, i.p. Sigma–Aldrich, St. Louis, USA) + scopolamine and served as positive control. Group IV and V received *C. sepiaria* 10 mg/kg and 40 mg/kg respectively + scopolamine.

The study lasted 16 days and all groups received the different pre-treatments for 16 days. From day 1 to day 10, animals only received daily administration of the different treatments. The behavioral tests began from day 11 for the Morris water maze and on the day12 for novel object recognition (NOR) and object location tasks (OLT). From day 11–16, Groups II, III, IV, and V, in addition to receiving the different treatments mentioned above, were co-treated with scopolamine (1 mg/kg, i.p., Sigma–Aldrich, St. Louis, USA) for six consecutive days. The various pre-treatments were administered 1 h prior to the behavioral tests, while scopolamine was administered 30 min prior to the behavioral tests.

The familiarisation phase of the novel object recognition (NOR) and object location tasks (OLT) were performed from day 12 to day 14 in a wooden box, before every scopolamine administration. The test phase of NOR and OLT was performed on day 15, while the MWM probe test was performed on day 16. One hour (1 h) after the MWM probe test, animals were sacrificed. Distilled water and *C. sepiaria* were administered by oral gavage.

### 2.4 Novel object recognition test

This test measures the ability to distinguish between new and familiar (previously encountered) objects. During the first three days, mice were habituated to set up by placing them in an empty wooden square box (40 ×40×45 cm) for a duration of 15 min. The sample phase and the test phase were both performed on the fourth day, the sample test being performed an hour before the test phase. The object used were two small identical wooden cubes and a wooden pyramid shape. During the sample stage, animals were individually exposed to the objects (wooden cubes) for a duration of 5 min. In the test stage, a new object (wooden pyramid shape) swapped one of the two familiar objects, and for a total duration of 5 min the exploration times of the novel and familiar objects were recorded. The objects were cleaned with ethanol 70% between testing. Animals which spent more time exploring the new object showed unharmed memory (Ngoupaye et al., 2020b).

### 2.5 Object-location task

Object-location task assesses spatial memory related to the hippocampus and measured the animal’s ability to discriminate the change in position of an object (Ennaceur et al., 1997). Two wooden cubes were used as the objects and were located in a wooden square box (40 × 40 × 45 cm). Mice were initially familiarized by introducing them individually in the wooden box without any object and allowed to explore for 15 min, for three successive days. In the sample stage on the fourth day, mice were individually placed in the wooden box containing two identical wooden cubes for 5 min. After a latent period of 30 min the test stage was started, it consisted of changing one object's position to a new location. The period spent exploring the objects at the familiar and new locations was taken for 5 min. Unharmed memory was supported by animals taking a longer time exploring the object in the new location (Antunes and Biala, 2012).

### 2.6 Morris water maze test

The Morris water maze is a circular pool (100 cm in diameter and 45 cm high) half filled with water (25 °C) and divided into four quadrants of equal area. A platform (6 cm wide and 29 cm high) was placed in one of the quadrants and immerged at 1 cm below the surface of the water making it invisible for the mouse. The test was conducted in two phases: the training phase during the first four days and the probe test on the 6th day with the 5th day being the resting day where animals were only receiving treatments and scopolamine without be submitted to the MWM test. Concerning the training phase, animals were individually introduced in the pool while facing the observer and the position of the platform was kept unchanged throughout the trials. Animals were then allowed to search for the platform for a maximum of 1 min; at the end of the 1 min, if the platform was not reached the animal was gently directed to the platform and allowed to stay on it for 20 s, before being removed from the pool. Concerning the probe test, the platform was removed from the pool and the animal was allowed to locate the position of the quadrant where the platform used to be, for a total duration of 1 min. The time spent in the quadrant containing the platform was measured, and the latency to reach the platform was measured and noted as the index of acquisition and learning (Ngoupaye et al., 2018).

### 2.7 Biochemical tests

#### 2.7.1 Homogenates preparation

Immediately after the last test (16th day), the animals were sacrificed by cervical dislocation, and each animal's brain was carefully isolated and their hippocampi collected. The hippocampus was grinded in a 0.1 M phosphate buffer containing 1% Triton-100X (P^H^ 7.4) (10% w/v) in a porcelain mortar to produce. The homogenates were later centrifuged for 15 min (3000 rpm), and the supernatant was collected for various assays (Ngoupaye et al. 2018).

#### 2.7.2 Assessment of antioxidant markers levels (reduced gluthathione (GSH), Malondialdehyde (MDA), Catalase activity (CAT)) in the hippocampus

##### 2.7.2.1 Measurement of GSH content

Tissue levels of reduced glutathione was determined by the method described by Ellman (1959). Briefly, in tubes containing 1.5 mL of the Ellman's reagent, 100 µL of the homogenate, and 100 µL of Phosphate buffer solution was added and the mixture was incubated at room temperature for one hour. The absorbance was read at 412 nm with a BIORAD spectrophotometer, SMART SPEC 3000 (USA). GSH tissue content was expressed as µmol/mL/mg.

##### 2.7.2.2 Measurement of Malondialdehyde (MDA) content

Lipid peroxidation was evaluated by determining the tissue levels of MDA. MDA was spectrophotometrically quantified using the thiobarbituric acid assay (Hritcu et al. 2011). 1 mL of 50% trichloroacetic acid in 0.1 M HCl and 1 mL of 26 mM thiobarbituric acid was added to a volume of 200 mL of homogenate and briefly mixed. The samples were incubated at 100º C for 20 min. The mixture was then centrifuged at 4000 rpm for 10 min to separate the organic layer. After the centrifugation, the absorbance was measured at 532 nm with a BIORAD spectrophotometer, SMART SPEC 3000 (USA). MDA levels were expressed as nmol/mg of tissue.

##### 2.7.2.3 Measurement of catalase activity

Catalase activity was determined by the colorimetric measurement of chromic acetate resulting from the reaction involving dichromate in the presence of acetic acid and hydrogen peroxide (H_2_O_2_) (Dimo et al. 2006). For the assay, a volume 375 µL phosphate buffer (PBS) was introduced into the test tube containing 25 µL of tissue supernatant. Subsequently, 100 µL of 50 mM hydrogen peroxide (H_2_O_2_) was introduced into the tube, and the reaction proceeded for 1 min, after which 1000 µL of the solution of 5% potassium dichromate + acetic acid was introduced into the reaction medium to stop the reaction. The tubes were then brought to boil for 10 min in a boiling water bath. After incubation, the tubes were cooled with running water, then the content of each tube (at least 1000 µL) was pipetted and read with a BIORAD spectrophotometer, SMART SPEC 3000 (USA) against the blank at 570 nm, and catalase activity was expressed as µM/min/mg of protein.

#### 2.7.3 Measurement of nitrite oxide

Hippocampi levels of nitrite oxide were determined according to the protocol described by Chang and Zhang (2017). This assay is based on the conversion of nitrate into nitrite by cadmium, followed by colour change upon mixture with freshly prepared Greiss reagent in acidic medium. 100 µL of Greiss reagent (0.1% N-(1-naphtyl) ethylene diamine dihidrochloride and 1% sulphanilamide in 5% orthophosphoric acid) in acidic medium was mixed with brain homogenates or sodium nitrite standard (100 µL), and incubated for 5 min at room temperature. Absorbance was measured using a BIORAD spectrophotometer, SMART SPEC 3000 (USA) against blank at 546 nm, and the results was expressed as µmol/mg of tissue.

#### 2.7.4 Evaluation of the effect of the root bark of *C. sepiaria* aqueous lyophilisate on acetylcholinesterase (AChE) activity

The amount AChE in the hippocampus was measured according to Ellman et al. (1961). A mixture of 1000 µL of buffer, 10 µL of 5, 50-dithiobis-2-nitrobenzoic acid (DTNB), and 25 µL acetylthiocholine iodide was introduced into Eppendorf tubes. The resulting solution was also used as a blank, and for the assay samples, 10 µL of the homogenates were added to the aforementioned solution, and the absorbance was read at 412 nm, using a BIORAD spectrophotometer, SMART SPEC 3000 (USA). Each reading was repeated three times, and the average of the 3 values was taken. The enzymatic activity was recorded for 5 min and expressed in nmol/min/mg of protein. The amounts of proteins were initially measured with a standard commercially available kit (Dutch Diagnostics).

### 2.8 Phytochemical quantification of the bioactive secondary metabolites of aqueous lyophilized extract of the root bark of *C. sepiaria*

#### 2.8.1 Determination of total phenolic content

Total phenolic content was determined by the method described by Ramde-Tiendrebeogo et al. (2012). The reagent consists of a mixture of phosphotungstic acid (H_3_PW_12_O_40_) and phosphomolybdic acid (H_3_PMo_12_O_40_). The reaction mixture in this assay consisted of 20 µL of extracts (2 mg/mL), 100 µL of Folin-Ciocalteu reagent (diluted 10-fold in water) and 80 µL of 20% sodium carbonate solution. The mixture was stirred and incubated in a water bath at 20 °C for 30 min, then the absorbance was measured with a microplate reader at 765 nm, against a blank containing distilled water. A calibration curve was plotted using gallic acid (gallic acid concentration ranging from 0.015 to 2 mg/mL). Results were expressed as milligram equivalent of gallic acid per gram of extract (mg GAE/g extract), averaged from triplicate measurements.

#### 2.8.2 Determination of tannins content

Tannins content was determined by the Folin-Ciocalteu method as described by Govindappa et al. (2011). Briefly, the reaction mixture in this assay consisted of 100 µL of extract (2 mg/mL), 500 µL of Folin-Ciocalteu reagent (diluted 10-fold in water), 1000 µL of 35% sodium carbonate solution, and 8.4 mL of distilled water. The mixture was stirred and incubated at room temperature for 30 min, then the absorbance was measured with a microplate reader at 700 nm. The results were expressed as milligram equivalent of tannic acid per gram of extract (mg TAE/g extract) averaged from triplicate measurements.

#### 2.8.3 Determination of total flavonoid content

The flavonoid content was determined following the method described by Chang et al. (2002) with some modifications using quercetin as the standard. Briefly, 500 µL of the extract (2 mg/mL) were mixed with 150 µL of 5% sodium nitrate (NaNO_2_) and incubated for 5 min at room temperature. Then, to the mixture were added 150 µL of 10% aluminum trichloride (AlCl_3_) followed 6 min later by the addition of 1 mL of 1 M sodium hydroxide (NaOH). The mixture was incubated for 15 min at room temperature, and the absorbance was read with a microplate reader at 510 nm against blank using a spectrophotometer. The total flavonoid content was calculated using the calibration curve of quercetin (0.1–1 mg/mL). The experiment was performed in triplicate. The total flavonoid content was expressed as mg quercetin equivalent per gram of extracts (mg QE/g extract) averaged from triplicate measurements.

### 2.9 Acute toxicity

The acute toxicity of the extract was evaluated according to the method described by the Organization for Economic Cooperation and Development (OECD) guideline 420 for acute toxicity (OECD, 2001). Briefly, twenty nulliparous mice of both sexes aged between 8 and 12 weeks, weighing 20–25 g were used. Animals were randomly divided into 2 groups, A and B of 10 animals each. Group A was considered the control group and was subdivided into two groups A1 for males and A2 for females, and received only distilled water (10 mL/kg) by oral gavage. Group B was subdivided into two groups B1 for males and B2 for females, and orally received a single administration of *C. sepiara* at 5000 mg/kg body weight (BW). The animals were deprived of food 24 h prior to the administration of both treatments. Immediately after treatments, animals were observed individually and continuously for 1 h, and then grouped into cages according to their sexes and treatment received, and observed intermittently for 4 h. Animals were further observed daily for a period of 7 days following treatments administration. Animals were weighed daily and observed for any sign of toxicity and death. Animals were observed for adverse effects, such as hypoactivity, asthenia, salivation, stool consistency, aggressiveness and locomotion. On the 8th day, animals were weighed and sacrificed. Organs such as the liver, kidneys, lungs, heart, spleen, and brain were collected, weighed, and observed macroscopically to detect any tissue alteration (OECD, 2001).

### 2.10 Statistical analysis

Statistical analysis was performed using Graph Pad Prism version 5.00 (San Diego, CA, USA). One-way ANOVA followed by the Newman-Keuls post hoc test was used and a Two-way ANOVA followed by the Bonferroni test when needed to analyze data. All data are presented as mean ± SEM per group. When the P < 0.05, the difference between groups was considered statistically significant.

## 3 Results

### 3.1 *C. sepiaria* aqueous lyophilisate improves the learning and memory process in the MWM

**Fig. 1:**
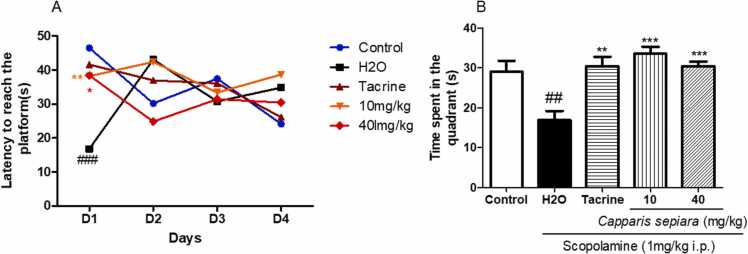
Effect of *of the root bark of C. sepiaria aqueous lyophilisate* on learning and memory assessed in the MWM. (A) Onset time to reach the hidden platform. (B) Time spent in the target quadrant. N = 7. All the data are expressed as mean ± SEM. *P < 0.05, * *p < 0.01, * **p < 0.001 compare to H2O, ###p < 0.001 compare to Control. Two-way ANOVA followed by the Bonferroni post-test. One-way ANOVA followed by the Newman Keuls post-test. Control: Vehicle, H2O: negative control, 10: *C. sepiaria* dose of 10 mg/kg, 40: *C. sepiaria* dose of 40 mg/kg.

Fig. 1a depicts the average latency to reach the hidden platform. Two-way ANOVA followed by the Bonferonni post-test revealed an interaction between groups during the four days of learning [F (12,84)= 2.741, p = 0.035]. On the first day, there was a significant increase of this latency in mice treated with Scopolamine alone compared to the control [F (1,12)= 5.530, p = 0.0366]. Animals treated with *C. sepiar*a (10 mg/kg and 40 mg/kg) significantly decreased the latency to reach the platform as compared to Scopolamine alone (H2O) [F (1,12)= 13.36, p = 0.0033], [F (1,12)= 5.121, p = 0.0471] respectively (Fig. 1a).

Fig. 1b shows the average time spent in the target quadrant during the probe test. There was a significant change in the time spent in the target quadrant between the different groups [F (4,34)= 8.879, p < 0.0001]. Animals treated with Scopolamine alone spent less time in the target quadrant as compared to the vehicle [F (2,20)= 8.007, p = 0.0033]. *C. sepiara* 10 mg/kg and *C. sepiara* 40 mg/kg prevented this effect [F (2,20)= 11.53, p = 0.0006] and [F (2,20)= 13.58, p = 0.0003], respectively.

### 3.2 *C. sepiaria* aqueous lyophilisate does not improve memory impairment on the Novel object recognition test

**Fig. 2:**
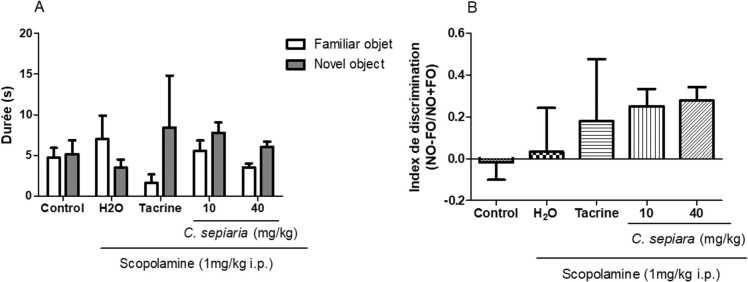
Effect of *of the root bark of C. sepiaria aqueous lyophilisate* in the Novel object recognition test. A- Represents the exploration duration between the two objects. B- Represents discrimination index. Each bar represents the mean ± SEM, N = 7. Two-way ANOVA followed by the Bonferroni post-test, One-way ANOVA followed by the Newman Keuls post-test. Control: Vehicle, H_2_O: negative control, 10: *C. sepiaria* dose of 10 mg/kg, 40: *C. sepiaria* dose of 40 mg/kg.

Fig. 2a shows the average time spent in the exploration of the familiar and novel object. There was no effect on the exploration duration observed on the novel and familiar object [F (4,60)= 1.165, p = 0. 335].

Fig. 2b represents the corresponding discrimination index between the familiar and the novel object. Accordingly, there was no effect on the discrimination index time done on the novel object and familiar object [F (2,20)= 0.584, p = 0.676].

### 3.3 *C. sepiaria* aqueous lyophilisate improves memory assessed on the location object test

**Fig. 3:**
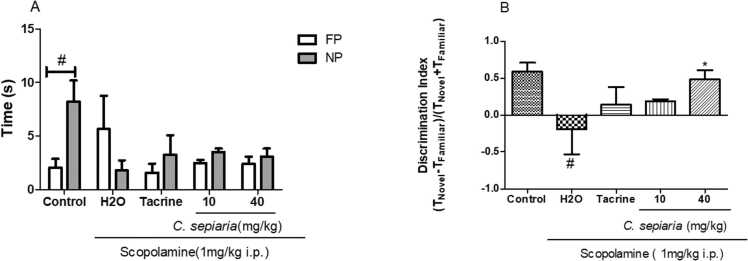
Effect of *of the root bark of C. sepiaria aqueous lyophilisate* on the object location test (OLT).

Fig. 3a below illustrates the average exploration time spent in the familiar and novel locations. Analysis revealed an interaction in the exploration time between the novel and the familiar position in the control group [F (4,58)= 3.105, p = 0.020].

The discriminating index corresponding to these exploration times is illustrated in Fig. 3b below. One way ANOVA shows that there was a scopolamine effect [F (3,27)= 3.464, p = 0.0320], when compared to the control group. Post hoc test showed that animal from the negative control showed a significant decreased of the discrimination index compared to the control [F (2,20)= 3.624, p = 0.0476]. This inability to discriminate objects by animals in the negative control group was prevented by *C. sepiara* (40 mg/kg) [F (2,20)= 3.829, p = 0.0412].

(A) Exploration duration. (B) Discrimination index. N = 7. All the data are expressed as mean ± SEM. #p < 0.05 compared to control (A), Control (B), *p < 0.05 compare to H2O. Two-way ANOVA followed by the Bonferroni post-test. One-way ANOVA followed by the Newman Keuls post-test. Control: Vehicle, H2O: negative control, 10: *C. sepiaria* dose of 10 mg/kg, 40: *C. sepiaria* dose of 40 mg/kg.

### 3.4 *C. sepiaria* aqueous lyophilisate improves antioxidant markers (glutathione (GSH), and catalase) and decreases pro-oxidant marker (Malondialdehyde (MDA)) and NO in the hippocampus

**Fig. 4:**
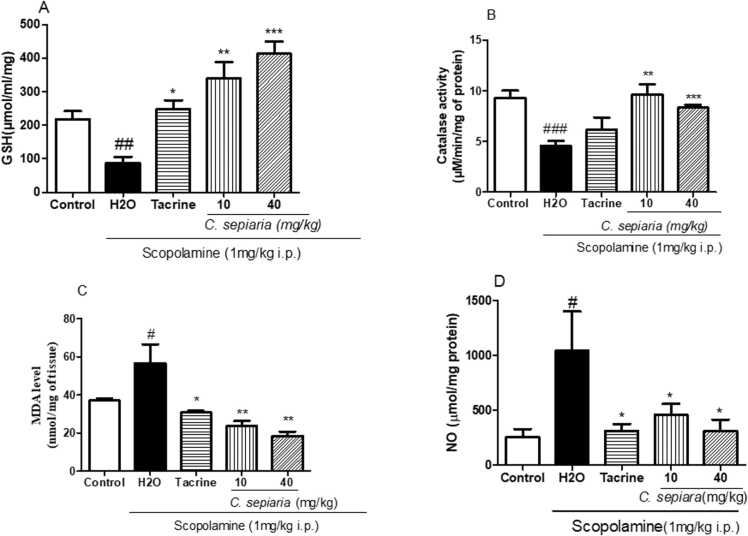
Effects of *of the root bark of C. sepiaria aqueous lyophilisate* on reduced glutathione (GSH), Malondialdehyde (MDA), Catalase activity and Nitrite oxide (NO) in the hippocampus. A: reduced glutathione (GSH) level, B: Catalase activity (MDA), C: Malondialdehyde (MDA) level. Each bar represents the means ± SEM N = 4, *p < 0.05 * *p < 0.01 * **p < 0.001 compared to H2O, #p < 0.01, ##p < 0.01, ###p < 0.001 compared to control. One-way ANOVA followed by the Newman Keuls post-test. Control: Vehicle, H2O: negative control, 10: *C. sepiaria* dose of 10 mg/kg, 40: *C. sepiaria* dose of 40 mg/kg.

Fig. 4 below represents the activity of *C. sepiara* on reactive oxygen species.

Fig. 4a shows that there was an overall treatment difference between groups in GSH levels [F(4,19) = 14.37, p < 0.0001]. There was a significant decreased in hippocampal GSH level in negative control mice compared to control mice [F(2,11) = 11.93, p = 0.0029]. This reduction in GSH level was inhibited when mice were pre-treated with *C. sepiara* (10 mg/kg and 40 mg/kg) before receiving Scopolamine [F(2,11) = 14.23, p = 0.0016] and [F(2,11) = 35.25, p < 0.0001] respectively.

The variation in the hippocampal level of catalase activity is highlighted in Fig. 4b below. One way analysis of variance reveals significant difference between groups [F(4,19) = 7.597, p = 0.0015]. Animals in the negative control group showed a significant decrease in catalase activity as compared to control group [F(2,11) = 21.36, p = 0.0004]. This decrease in catalase activity in the negative group was corrected in animals pre-treated with *C. sepiara* (10 mg/kg and 40 mg/kg) [F(2,11) = 16.20, p = 0.0010] and [F(2,11) = 22.50, p = 0.0003] respectively.

Fig. 4c represents the changes in the levels of MDA in the hippocampus following treatments. There was a significant difference between groups [F(4,19) = 10.17, p = 0.0003]. When compared to animal which received distilled water prior to scopolamine administration, animals which were pre-treated with *C. sepiara* (10 mg/kg and 40 mg/kg) depicted a significant reduction in MDA level [F(2,11) = 8.082, p = 0.0098] and [F(2,11) = 11.39, p = 0.0034] respectively.

Fig. 4d depicts how the levels of NO in the hippocampus varies with different treatments. Scopolamine-treated animals showed a significant increase in NO level [F(2,11) = 4.412, p = 0.0462]. This increase in NO levels was stopped by *C. sepiara* (10 mg/kg and 40 mg/kg) as animals treated with the extract showed a significant reduction in NO levels compared to animals treated with distilled water [F(4,19) = 3.315, p = 0.0341].

### 3.5 *C. sepiaria* aqueous lyophilisate decreases AChE activity in the hippocampus

**Fig. 5:**
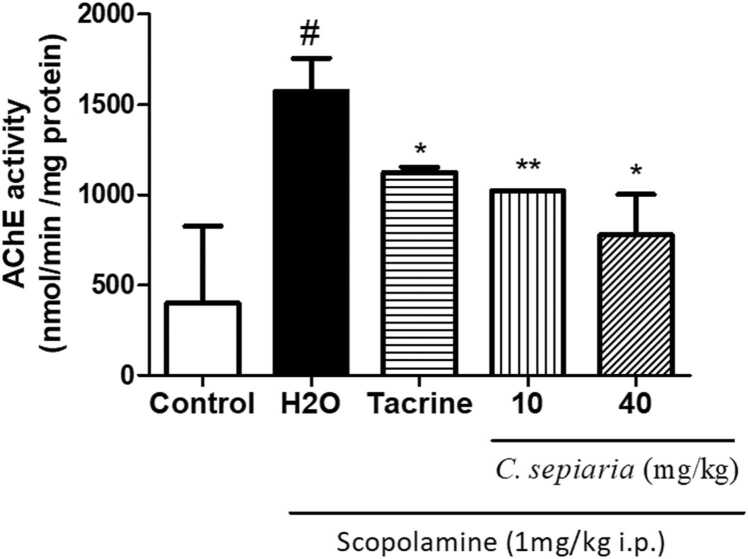
Effects of *C. sepiaria* evaluated on acetylcholinesterase activity. Each bar represents the mean ± SEM, N = 4. *p < 0.05 * *p < 0.01 compare to H2O, #p < 0.05 compared to Control. One-way ANOVA followed by the Newman Keuls post test Control: Vehicle, H2O: negative control, 10: *C. sepiaria* dose of 10 mg/kg, 40: *C. sepiaria* dose of 40 mg/kg.

Fig. 5 shows how AChE’s activity varies with various treatments. Analysis reveals that the treatment affected the AChE’s activity [F(4,19) = 3,.655, p = 0.0286]. AChE’s activity was significantly increased in the negative control group [F(2,11) = 4396, p = 0.046]. This effects was significantly reversed when animals were pretreated with *C. sepiara* (10 mg/kg and 40 mg/kg) [F(2,11) = 9130, p = 0.0068], and [F(2,11) = 7667, p = 0.0114] respectively.

### 3.6 Quantitative phytochemistry of the aqueous lyophilisate of the root bark of *C. sepiaria*

**Fig. 6:**
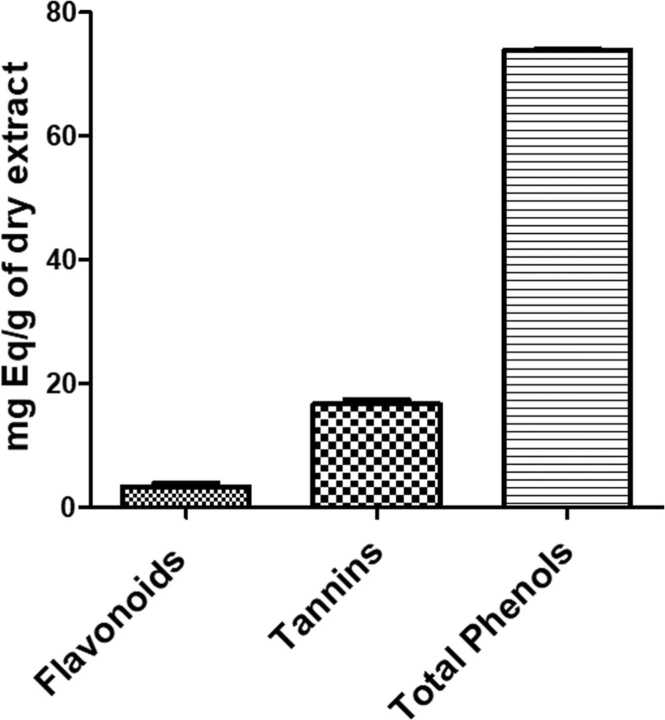
*Quantitative phytochemistry of the aqueous lyophilisate of the root of C. sepiaria*.

The aqueous lyophilisate of *C. sepiaria*’s roots contained: 3.37 ± 0.54 mg QE/g of flavonoids, 16.66 ± 0.73 mg TAE/g of tannins and 73.8 ± 0.33 mg/kg GAE/g of total phenols (Fig. 6).

Data expressed as mean ± SEM; n = 3.

### 3.7 Acute toxicity tests

#### 3.7.1 *C. sepiaria* aqueous lyophilisate shows less toxicity on mice

Behavioral studies and macroscopic observations following acute toxicity revealed no sign of toxicity nor mortality neither in mice which received distilled water as treatment nor in those who received *C. sepiaria* at the dose 5000 mg/kg (Table 1).

**Table 1:** Effects of the *root bark of C. sepiaria aqueous lyophilisate* on animal behavior and mortality.

*Parameters*	*Sex*	*Distilled water*	*C. sepiaria* *(5000 mg/kg)*
Asthenia	Males Females	- -	- -
Salivation	Males Females		- -
Hypoactivity	Males Females	- -	- -
Pain sensitivity	Males Females	N N	N N
Sensibility to noisy	Males Females	N N	N N
Aggressiveness	Males Females	N N	N N
Locomotion	Males Females	N N	N N
Stool consistency	Males Females	G G	G G
Mortality	/	0%	0%

N = 5. N: Normal, G: Granule.

#### 3.7.2 *C. sepiaria* aqueous lyophilisate does not altered animal’s body weight

No significant difference in the variation of the body weight was recorded between the animals treated with distilled water and those which received the different extracts (Table 2).

**Table 2:** *Effects of the aqueous lyophilisate of the root bark of C. sepiaria on body weight of animals*.

Parameters	Sex	Body weight (g) Day 1 Day 7		Mass variation (%)
Distilled water (10 mL/kg)	Males Females	25.60 ± 3.83 23.20 ± 1.47	28.40 ± 4.41 24.06 ± 2.23	15.63% 19.46%
*C. sepiaria* (5000 mg/kg)	Males Females	27.00 ± 4.56 21.20 ± 1.33	29.00 ± 4.60 25.60 ± 1.62	8.11% 13.86%

Data are represented as mean ± SEM, N = 5, Two-way ANOVA followed by Bonferroni post-test

### 3.8 *C. sepiaria* aqueous lyophilisate does not alter the relative masses of vital organs of animals

**Fig. 7:**
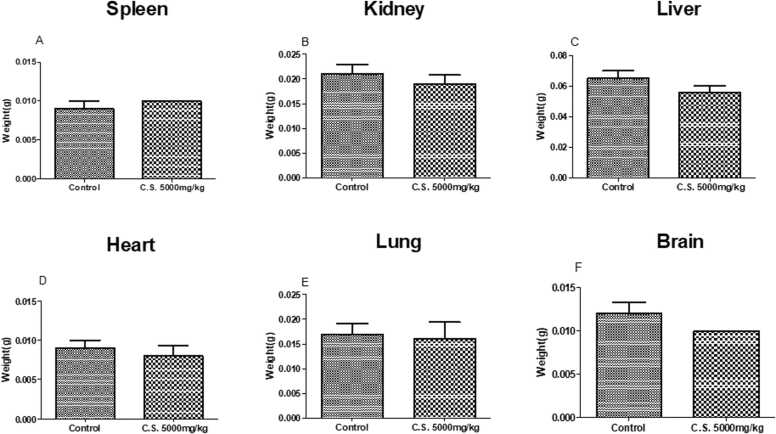
The relative weight of animal’s vital organs (spleen, liver, kidneys, heart, lungs and brain). Each bar represents the mean ± SEM, N = 10. Student t test. C.S. 5000 mg/kg: *C. sepiaria* 5000 mg/kg.

Fig. 7 depicts the relative weight of the animal's vital organs (spleen, liver, kidneys, heart, lungs, and brain). No significant difference was observed in the weight of these vital between the group of animals treated with *C. sepiaria* (5000 mg/kg) and those treated with distilled water (P > 0.05).

## 4 Discussion

The study aimed at assessing the anti-amnesic effect of *Capparis sepiaria* (*C. sepiaria*) on mice induced by Scopolamine. The Morris water maze test has been extensively used to assess spatial memory (Vorhees and Williams, 2006). The above results show that pre-treatment with the lyophilisate improved memory recall during the probe test, suggesting that scopolamine-induced memory alteration was corrected. These results are similar to those obtained by who reported that there was an improvement of memory in mice pre-treated with *Drymaria cordata* and rat pre-treated with *Gladiolus dalenii* as assessed by the Morris water maze test. The effects of *C. sepiaria* on cognitive functions were further assessed by the Novel object recognition test (NORT) and object location test (OLT). Deficits in episodic memory were evaluated in the NORT. It follows that the lyophilisate did not show any effect on the NORT as there was no difference between the untreated group exposed to scopolamine and the groups pre-treated with *C. sepiara*, suggesting that *C. sepiara* was not able to restore scopolamine-induced memory episodic impairment. However, the extract was able to restore spatial memory. Indeed, the lyophilisate of *C. sepiara* increased the index of discrimination between the novel location and the old location of the object compared to the negative control. Undoubtedly, the OLT is a simple and effective test that provides a measure of hippocampus-dependent spatial memory (Vogel-Ciernia and Wood, 2014), and has been widely used to examine the effects of various pharmacological treatments on brain damage (Vogel-ciernia and wood, 2014). It is established that the rodents have a natural propensity to explore a new object (Ennaceur et al., 1997). Given the specificity of the MWM and the OLT in evaluating spatial memory and hippocampal structure, the results obtained in this study following MWM, OLT and NORT-evaluated cognitive functions reveals that *C. sepiaria* alleviates scopolamine-induced memory deficit by a specific interaction with hippocampal associated task, especially by improving spatial memory.

Oxidative stress plays an important role in memory deficit induced by scopolamine (Budzynska et al., 2015; Haider et al., 2016), thus, in an attempt to elucidate the possible mechanisms of action through which *C. sepiaria* restores spatial memory following scopolamine administration, the antioxidant capacity of the aqueous lyophilisate of *C. sepiaria* was assessed. Previous studies have shown the ability of scopolamine to induce oxidative stress by means of brain lipid peroxidation and thus decreasing the level and activity of antioxidant species such as reduced glutathione respectively (Ngoupaye et al., 2017). The implication of lipid peroxidation in mechanisms leading to cell injury in animal models are well established, making it a suitable indicator of oxidative stress in cell tissues (Liu et al. 2017). Polyunsaturated fatty acids derived from lipid peroxides are unstable compound and break down to form a series of complex compounds such as MDA. All these observations were evidenced in this study as, scopolamine has significantly decreased the levels of reduced glutathione and catalase activity, and increased the level of malondialdehyde as seen in mice of the negative control. The ability of *C. sepiaria* to reverse the release of reactive oxygen species by scopolamine administration suggests that it possesses antioxidant activities and is able to prevent damages such as peroxidation. The nitrite oxide scavenging ability of *C. sepiaria* extract further expand its role as potent antioxidant as NO is known to be a free radical derived from reactive nitrogen species and in its free form is harmful to biomolecules and has a deleterious effect on neurons (Liu et al., 2017). These observed antioxidant effects could be attributed to second metabolites such as Phenols, flavonoids and tannins which are contained in the roots of *C. sepiaria*. Indeed, phenolic compounds especially polyphenol has been reported to show antioxidant property by preventing lipid peroxidation. Flavonoids have the propensity to donate the hydrogen atom attached to the aromatic ring structures which would result in the reduction of Fe^3+^ to Fe^2+^ which serves as a significant antioxidant indicator (Gulcin, 2020). Together with flavonoids, tannins are members of the family of phenolic compounds, with high molecular weight, and whose antioxidant properties have been demonstrated as well (Hussain et al., 2011). Thus, the high antioxidant activity of *C. sepiaria* could be due to its total phenolic content.

AChE is a key enzyme that cleaves synaptic ACh and thus regulates cholinergic functions. Cognitive impairment could be derived from acetylcholine (Ach) deﬁciency caused by excessive AChE activity (), and studies suggests that inactivation of AChE activity improves memory impairment (He et al., 2015). In humans and animals, the hippocampus has been reported to play an essential role in encoding and retrieving spatial memory processes (Ngoupaye et al., 2022). Consequently, damage and/or functional disturbances in this brain region are associated with severe cognitive impairments. Our results show that animals treated with Scopolamine showed an increased hippocampal AChE activity. This increase was significantly decreased when animals were pre-treated with *C. sepiara,* reflecting the ability of the aqueous lyophilizate to restore hippocampal cholinergic function.

According to the globally harmonized classification system (GHS), the aqueous extract of *C. sepiaria* would be classified in category 5 of substances with relatively low acute toxicity (OECD, 2001) as no mortality was recorded, and neither significant effect was observed in the behaviour tests nor on the relative mass of the vital organs. Toxicity studies with the ethanolic extracts of the plant performed by Rajesh et al. (2010) did not reveal any toxicity sign in animals and also did not show any behavioural change from 100 mg/kg/b.w. up to 5000 mg/kg/b.w. These results suggest that this extract has no toxic effects and is well tolerated in mice.

In conclusion, *C. sepiaria* shows anti-amnesic properties, by preventing cognitive impairment induced by scopolamine. Part of these properties is mediated through oxidative stress and acetylcholinesterase inhibitions. Moreover, it is not toxic on acute toxicity model. The present results provide, at least in part, some understanding of the scientiﬁc rationale supporting the use of *C. sepiaria* in traditional medicine.

## Ethical statements

The animal used in the experimental procedure was approved under the guidelines of the Cameroonian bioethics committee (reg N°. FWA-IRB00001954) and in accordance with NIH- Care and Use of Laboratory Animals manual. Efforts were also made to minimize animals' suffering and to decrease the number of animals utilized in the experiment.

## Funding

The authors declare that no funds, grants, or other support were received during the preparation of this manuscript.

## CRediT authorship contribution statement

**Francis Bray Yassi** co-designed the experiments, conducted the laboratory trials, data analysis and manuscript writing as part of its PhD’s thesis; **Gwladys Temkou Ngoupaye** designed the work and supervised the experiments, data analysis and manuscript writing; **Tatiana Diebo Kom, Gabriella Dongmo Tonleu**, **Maxwell Blesdel Adassi** and **Aurelien Fossueh Foutsop** gave support for behavioural and biochemical tests; **Elisabeth Ngo Bum** supervised the work, manuscript writing and gave general advices.

## Competing interest

The authors have no relevant financial or non-financial interests to disclose.
